# Annual acknowledgement of manuscript reviewers

**DOI:** 10.1186/1423-0127-21-9

**Published:** 2014-02-12

**Authors:** Wen-Chang Chang

**Affiliations:** 1 Taipei Medical University, Taipei, Taiwan

## Abstract

**Contributing reviewers:**

The editors of *Journal of Biomedical Science* would like to thank all our reviewers who have contributed to the journal in Volume 20 (2013).

## 

Sazaly AbuBakar

Malaysia

Jalal Alizadeh

Iran

Cresio Alves

Brazil

Mutsuki Amano

Japan

Michael N Antoniou

United Kingdom

Joseph Audie

United States of America

Alimohammad Bahrami

Iran

Yoshio Bando

Japan

Brian Bandy

Canada

Debabrata Banerjee

United States of America

Anna Barsukova

United States of America

Joyoti Basu

India

Richard Bell

United States of America

Tone Berge

Norway

Ben Berkhout

Netherlands

Jacques Bertoglio

France

Jon Bible

United Kingdom

Andrea Boggild

Canada

Alexandre M.J.J. Bonvin

Netherlands

Hossein Borghaei

United States of America

Albert Breier

Slovakia

Vincent Burrus

Canada

Tai-Lung Cha

Taiwan

Julie Y.H. Chan

Taiwan

Lo Kong Chan

Hong Kong

Nei-Li Chan

Taiwan

Samuel H.H. Chan

Taiwan

Yoke-Fun Chan

Malaysia

Alice Y.W. Chang

Taiwan

Chawnshang Chang

United States of America

Chungming Chang

Taiwan

I-Shou Chang

Taiwan

Jer-Wei Chang

Taiwan

Ming-Fu Chang

Taiwan

Nan-Shan Chang

Taiwan

Tsuchung Chang

Taiwan

Wen-Chang Chang

Taiwan

Wun-Shaing Wayne Chang

Taiwan

Yu-sun Chang

Taiwan

Fadi Charchar

Australia

Ann Chen

Taiwan

Ben-Kuen Chen

Taiwan

Cheng-Nan Chen

Taiwan

Chia-Hsiang Chen

Taiwan

Chien Hung Chen

Taiwan

Chuan-Mu Chen

Taiwan

Chun-Ming Chen

Taiwan

Emily Chen

United States of America

Hsing I Chen

Taiwan

Hua-Chien Chen

Taiwan

Huey-Ling Chen

Taiwan

Jaw-Wen Chen

Taiwan

Jin-Chung Chen

Taiwan

Jin-Jer Chen

Taiwan

Jun Chen

United States of America

Kuen-Feng Chen

Taiwan

Mei-Ling Chen

Taiwan

Pei-Yu Chen

United States of America

Show-Li Chen

Taiwan

Ya-Wen Chen

Taiwan

Yi-Jen Chen

Taiwan

Naichen Cheng

Taiwan

Ya-Hui Chi

Taiwan

Bor-Luen Chiang

Taiwan

Yung-Hsiao Chiang

Taiwan

Chiang Chien

Taiwan

Sarin Chimnaronk

Thailand

Chang-Fang Chiu

Taiwan

Ing-Ming Chiu

Taiwan

Jeng-Jiann Chiu

Taiwan

Chang Hoon Cho

United States of America

Pele Chong

Taiwan

Tz-Chong Chou

Taiwan

Yu-Ting Chou

Taiwan

Lu-Ping Chow

Taiwan

Shuang-En Chuang

Taiwan

Yao-Chung Chuang

Taiwan

Chih-Pin Chuu

Taiwan

Lori Coburn

United States of America

John Coligan

United States of America

Yuan-Ji Day

Taiwan

Rosa del Angel

Mexico

Win-Ping Deng

Taiwan

Umesh Deshmukh

United States of America

Philippe Despres

France

Jim Deuchars

United Kingdom

Michele Di Mascio

United States of America

Jean-Luc Dreyer

Switzerland

Matthias Duerst

Germany

Malcolm Duthie

United States of America

Ralf Einspanier

Germany

Ann-Kathrin Eisfeld

United States of America

john Clive Ellory

United Kingdom

Hoda Elsayed

Egypt

Audrey Esclatine

France

Kang Fang

Taiwan

Saeid Fathi

Iran

Trevor Ferguson

Jamaica

Dan Frenkel

Israel

Judith Friesenhagen

Germany

Wen-Mei Fu

Taiwan

Masahiro Fujimuro

Japan

Yasuhisa Fukui

United Kingdom

Fukumi Furukawa

Japan

Guadalupe García-Elorriaga

Mexico

Jose Luis Garcia-Perez

Spain

Patricio Gariglio

Mexico

Po-Wu Gean

Taiwan

Luo-Ping Ger

Taiwan

Payam Gharibani

United States of America

Gargi Ghosal

United States of America

Peter Goon

United Kingdom

Sabine Grösch

Germany

Esther Grueso

Germany

Jih-Hwa Guh

Taiwan

Scott Halstead

United States of America

Peidong Han

United States of America

Youssef Hatem

Egypt

Satoshi Higa

Japan

Yoshitaka Hirooka

Japan

Ching-Liang Ho

Taiwan

Tomohisa Horibe

Japan

Yoshiyuki Horio

Japan

Peter Hornsby

United States of America

George Hsiao

Taiwan

Ching-Hua Hsieh

Taiwan

Hsing-Pang Hsieh

Taiwan

Po-Shiuan Hsieh

Taiwan

Sen-Yung Hsieh

Taiwan

Song-Chou Hsieh

Taiwan

Tsai-Yuan Hsieh

Taiwan

Chin Hsu

Taiwan

Ching-Sheng Hsu

Taiwan

Chung Hsu

Taiwan

Hsin-Ling Hsu

Taiwan

Kuei-Sen Hsu

Taiwan

Ping-Ning Hsu

Taiwan

Kuo-Tai Hua

Taiwan

Eagle Yi-Kung Huang

Taiwan

Shih-Ming Huang

Taiwan

Shiu-Feng Huang

Taiwan

Tur-Fu Huang

Taiwan

Xi Huang

China

Xupei Huang

United States of America

Yi-Tsau Huang

Taiwan

Jan-Jong Hung

Taiwan

Li-Man Hung

Taiwan

Shiaw-Min Hwang

Taiwan

Bartholomew Ibeh

Nigeria

Reiko Inagi

Japan

William Jackson

United States of America

Rienk Jeeninga

Netherlands

Seng-Feng Jeng

Taiwan

Yung-Ming Jeng

Taiwan

Ching-Chuan Jiang

Taiwan

Xinguo Jiang

United States of America

Yun-Jin Jiang

Taiwan

Zheng Jiang

United States of America

MCecilia Johnson

Chile

Yuh-Shan Jou

Taiwan

Hsueh-Fen Juan

Taiwan

Suh-Hang Juo

Taiwan

Marian Kacerovsky

Czech Republic

Shinji Kamada

Japan

Hong-Yo Kang

Taiwan

Bulent Karagoz

Turkey

Masaaki Kawai

Japan

Ushio Kikkawa

Japan

Akira Kikuchi

Japan

Takuya Kishi

Japan

Magdalena Krol

Poland

Yoshiaki Kubota

Japan

John Kung

Taiwan

Calvin Kunin

United States of America

Cheng-Chin Kuo

Taiwan

Yu-Min Kuo

Taiwan

Sandro La Vignera

Italy

Chyong Huey Lai

Taiwan

Jenn-Haung Lai

Taiwan

Hsien-Yuan Lane

Taiwan

Gary Latham

United States of America

Chien-Kuo Lee

Taiwan

Hsuan-Shu Lee

Taiwan

Kai-Fai Lee

China

Ming-Shyue Lee

Taiwan

Oscar Lee

Taiwan

T–C Lee

Taiwan

Tzong-Shyuan Lee

Taiwan

Wei-Chia Lee

Taiwan

Yi-Hsuan Lee

Taiwan

Guey-Jen Lee-Chen

Taiwan

Steve Leu

Taiwan

Yuk Leung

Taiwan

Hua-Jung Li

Taiwan

Chun-Jen Liao

Taiwan

Jyh-Fei Liao

Taiwan

Cheng-Wen Lin

Taiwan

Chih-Lin Lin

Taiwan

Ching-Hsuan Lin

Taiwan

Ching-Yuang Lin

Taiwan

Chin-Tarng Lin

Taiwan

Chiou-Feng Lin

Taiwan

Kai-Ti Lin

Taiwan

Kuo-I Lin

Taiwan

Kwang-Huei Lin

Taiwan

Su-Fang Lin

Taiwan

Teng-Nan Lin

Taiwan

Wen-Chang Lin

Taiwan

Ya-Wen Lin

Taiwan

Yu-Tsan Lin

Taiwan

Jun-Yang Liou

Taiwan

Chia-Chyi Liu

Taiwan

Chih-Pin Liu

United States of America

Fu-Tong Liu

United States of America

Lianxing Liu

United States of America

Shing-Hwa Liu

Taiwan

Shufeng Liu

United States of America

Yan Liu

United States of America

Yi-Wen Liu

Taiwan

Zhenguo Liu

United States of America

Hsiu-Jung Lo

Taiwan

Szecheng Lo

Taiwan

Chun-Yi Lu

Taiwan

Ru-Band Lu

Taiwan

Yong Lu

United States of America

Kerstin Luhn

United Kingdom

Yongjie Ma

United States of America

Suresh Mahalingam

Australia

Sekhar Majumdar

India

Daniel Malamud

United States of America

Gathsaurie Malavige

Sri Lanka

Mark Mamula

United States of America

Isao Matsuura

Taiwan

Meei-Ling Sheu

Taiwan

Yong Miao

United States of America

Ryo Miyazaki

Switzerland

Dave Morgan

United States of America

Ikuo Morita

Japan

Dafne Müller

Germany

Mariappan Muthuchamy

United States of America

Hironori Nakagami

Japan

Akio Nakashima

Japan

Changlong Nan

United States of America

Purushottam Narute

United States of America

Britto Nathan

United States of America

Yen-Hsuan Ni

Taiwan

Barbara Niemeyer

Germany

Sansanee Noisakran

Thailand

Lisa Nonaka

Japan

Shigefumi Okamoto

Japan

Makoto Orisaka

Japan

Raoul Orvieto

Israel

Haydar Ozpinar

Turkey

Li-Mei Pai

Taiwan

Chunliu Pan

United States of America

Harshal Pandve

India

Luther Lalit Kumar pmeswaran Grace

Sweden

Guey Chuen Perng

Taiwan

Brunella Posteraro

Italy

Howard Prentice

United States of America

Yan Qin

United States of America

Jian-Tai Qiu

Taiwan

Jacob Raber

United States of America

Swaraj Rajkhowa

India

Marc Ramael

Belgium

Azra Raza

United States of America

Hoon Ryu

United States of America

Fahri Saatcioglu

Norway

Tetsushi Sadakata

Japan

Leticia Santos

Mexico

Sankar Sanyal

India

D. Scott Schmid

United States of America

Daniel Scott

France

Aleksi Sedo

Czech Republic

James Sham

United States of America

Soroush Sharbati

Germany

Chia-Ning Shen

Taiwan

Meng-Ru Shen

Taiwan

Wen Shen

United States of America

Shubhada Shenai

India

Joen-Rong Sheu

Taiwan

Shwu-Jiuan Sheu

Taiwan

Guey-Yueh Shi

Taiwan

Jianjun Shi

United States of America

Shine-Gwo Shiah

Taiwan

Kun-Ruey Shieh

Taiwan

Sheau-Yann Shieh

Taiwan

Hsiu-Ming Shih

Taiwan

Neng Yao Shih

Taiwan

Hao-Ai Shui

Taiwan

Jia-Fwu Shyu

Taiwan

Kou-Gi Shyu

Taiwan

Duncan R. Smith

Thailand

Jiangping Song

China

Néstor Soria

Argentina

Jesus Aldudo Soto

Spain

James St John

Australia

Blair Strang

United States of America

Chien-Wei Su

Taiwan

Ih-Jen Su

Taiwan

Jin-Yuan Su

Taiwan

Jun-Han Su

United States of America

Ming-Jai Su

Taiwan

Tung-Hung Su

Taiwan

Jau-Ling Suen

Taiwan

Kaoru Sugasawa

Japan

Dengyun Sun

United States of America

Peifang Sun

United States of America

Man-Sun Sy

United States of America

Ruth Tachezy

Czech Republic

You-Lin Tain

United States of America

Bertrand Tan

Taiwan

Tse-Hua Tan

Taiwan

Toshihiro Tanaka

Japan

MI-Hua Tao

Taiwan

Rui Tao

United States of America

Julia Tchou

United States of America

Athmaram TN

India

Kenneth Tsai

United States of America

Kun-Chih Kelvin Tsai

Taiwan

Shaw-Jenq Tsai

Taiwan

Shih-Feng Tsai

Taiwan

Ting-Fen Tsai

Taiwan

Ching-Jiunn Tseng

Taiwan

Shun-Fen Tzeng

Taiwan

Natsuo Ueda

Japan

Arie van der Ende

Netherlands

H Rogier van Doorn

Viet Nam

Thomas van Groen

Finland

Sudhakar Veeranki

United States of America

Thirunavukkarasu Velusamy

United States of America

Ivan Vickers

Jamaica

Carlijn Voermans

Netherlands

Tamara Vorobjova

Estonia

Feng-Sheng Wang

Taiwan

Hsei-Wei Wang

Taiwan

Jaw-Yuan Wang

Taiwan

Jia-Yi Wang

Taiwan

Ju-Ming Wang

Taiwan

Jung-Pan Wang

Taiwan

Li-Yu Wang

Taiwan

Yi-Ching Wang

Taiwan

Naoki Watanabe

Japan

Haiming Wei

China

Jenny Wei

United States of America

Yau-Huei Wei

Taiwan

Xiaoyun Wen

United States of America

David Williams

United States of America

Viroj Wiwanitkit

Thailand

Chih-Shung Wong

Taiwan

Bin-Nan Wu

Taiwan

Chin-Chen Wu

Taiwan

Gwo-Jang Wu

Taiwan

Han-Chung Wu

Taiwan

Jen-Leih Wu

Taiwan

Jinhua Wu

United States of America

Jiunn-Jong Wu

Taiwan

Kay LH Wu

Taiwan

Kou-Juey Wu

Taiwan

Ming-Shiang Wu

Taiwan

T.-C. Wu

United States of America

Yang-Chang Wu

Taiwan

Betty Wu-Hsieh

Taiwan

Hongyu Xue

United States of America

Munekazu Yamakuchi

Japan

Ryo Yamamoto

Japan

Seiya Yamayoshi

Japan

Bo-Shiun Yan

Taiwan

Yu-Ting Yan

Taiwan

Bei-Chang Yang

Taiwan

Chang-Hao Yang

Taiwan

Chuen-Mao Yang

Taiwan

Hung-Chih Yang

Taiwan

Jenq-Lin Yang

Taiwan

Muh-Hwa Yang

Taiwan

Rong-Sen Yang

Taiwan

Wei-Shiung Yang

Taiwan

Yun-Liang Yang

Taiwan

Mumtaz Yaseen

United States of America

Sheng Ye

China

Hung-I Yeh

Taiwan

Shiou-Hwei Yeh

Taiwan

B. Linju Yen

Taiwan

Yin-lu Ding

China

Junko Yoshida

Japan

Jau-Song Yu

Taiwan

Jiashing Yu

Taiwan

Chiou-Hwa Yuh

Taiwan

Jiangwei Zhang

United States of America

Yong Zhang

China

Zhenyu Zhang

United States of America

